# Efficacy of autologous blood patch injection for pneumothorax rate after CT-guided percutaneous transthoracic lung biopsy: a systematic review and meta-analysis

**DOI:** 10.1186/s13019-024-02781-0

**Published:** 2024-06-14

**Authors:** Xin Chen, Yungui Bian, Hai Li, Shurong Li, Zhaowen Shi, Yunping Zhao, Haibin Li, Yanlong Yang

**Affiliations:** 1 Department of General Surgery, The People’s Hospital of Fengqing, Lincang, 675900 PR China; 2 Department of Thoracic Surgery, The People’s Hospital of Fengqing, Lincang, 675900 PR China; 3 Department of Stomatology, The People’s Hospital of Fengqing, Lincang, 675900 PR China; 4 Department of Clinical Laboratory, The People’s Hospital of Fengqing, Lincang, 675900 PR China; 5 Department of Thoracic Surgery I, The First Affiliated Hospital of Kunming Medical University, No.295 Xichang Road, Wuhua District, Kunming, Yunnan Province 650032 PR China

**Keywords:** Autologous blood Patch, CT-guided percutaneous transthoracic lung biopsy, Pneumothorax

## Abstract

**Background:**

Pneumothorax is the most frequent complication after CT-guided percutaneous transthoracic lung biopsy (CT-PTLB). Many studies reported that injection of autologous blood patch (ABP) during biopsy needle withdrawal could reduce the pneumothorax and chest tube insertion rate after CT-PTLB, but the result is debatable. The aim of this systematic review and meta-analysis is to synthesize evidence regarding the efficacy of ABP procedure in patients receiving CT-PTLB.

**Methods:**

Eligible studies were searched in Pubmed, Embase and Web of Science databases. The inclusion criteria were studies that assessed the relationship between ABP and the pneumothorax and/or chest tube insertion rate after CT-PTLB. Subgroup analyses according to study type, emphysema status and ABP technique applied were also conducted. Odds ratio (OR) with 95% confidence interval (CI) were calculated to examine the risk association.

**Results:**

A total of 10 studies including 3874 patients were qualified for analysis. Our analysis suggested that ABP reduced the pneumothorax (incidence: 20.0% vs. 27.9%, OR = 0.67, 95% CI = 0.48–0.66, *P* < 0.001) and chest tube insertion rate (incidence: 4.0% vs. 8.0%, OR = 0.47, 95% CI = 0.34–0.65, *P* < 0.001) after CT-PTLB. Subgroup analysis according to study type (RCT or retrospective study), emphysema status (with or without emphysema), and ABP technique applied (clotted or non-clotted ABP) were also performed and we found ABP reduced the pneumothorax and chest tube insertion rate in all subgroups.

**Conclusions:**

Our study indicated that the use of ABP was effective technique in reducing the pneumothorax and chest tube insertion rate after CT-PTLB.

**Supplementary Information:**

The online version contains supplementary material available at 10.1186/s13019-024-02781-0.

## Introduction

CT-guided percutaneous transthoracic lung biopsy (CT-PTLB) is a well-established procedure to obtain tissue from pulmonary lesions [1]. Pneumothorax is the most frequent complication, ranging from 18.8 to 45% and the chest tube insertion rate ranging from 4.3 to 20% according to report from several societies’ guidelines [2]. This increases the pain, the length of hospitalization and the cost of patients. Some factors may increase the risk of pneumothorax such as emphysematous lungs, larger calibre guide/needles, bulla crossed, fissure crossed, multiple pleural punctures, smaller lesions, lesions without pleural contact and deeper lesions [2]. Some technologies have been proposed to reduce the incidence of pneumothorax. For example, deep expiration and breath hold during needle extraction, biopsy-down position (ipsilateral decubitus position), and a rapid needle-out patient-rollover technique [2, 3].

Sealing the lung biopsy tract with substances such as saline, blood, gelatin sponge slurry, or fibrin glue is an efficient technique to reduce the risk of pneumothorax development after the needle is withdrawn [3–5]. When compared with other sealant, autologous blood patch (ABP) is simple, fast, economical and efficacy [6]. In recent years, many studies evaluate the efficacy of ABP in reducing the pneumothorax incidence after CT-PTLB. However, the conclusion was not consistent. Some studies reported ABP could significantly reduce the pneumothorax and chest tube insertion rate [7], but some study could not draw such conclusion [8]. Due to the limited sample size and static power in individual study, a meta-analysis is necessary to evaluate safety and efficacy of ABP procedure in patients receiving CT-PTLB.

## Methods

A comprehensive literature search was conducted in the databases of PubMed, EMBASE, Web of Science and the Chinese National Knowledge Infrastructure (CNKI). The last search time was May 30, 2023. The following search terms were used to identify studies: (biopsy OR fine-needle OR fine needle aspiration OR FNA) AND (transcutane OR percutane OR transthoracic OR computerized tomography-guided OR CT-guided) AND (lung cancer OR lung neoplasms OR nodule OR pulmonary OR lung) AND (blood patch OR autologous blood patch OR blood patching OR blood clot OR autologous blood injection). Furthermore, references of retrieved articles and reviews were manually screened for additional studies.

### Inclusion and exclusion criteria

The inclusion criteria were applied to identify the eligible studies: (1) comparative studies compared ABP with control; (2) at least one of the following information was reported: pneumothorax rate or chest tube insertion rate; (3) to provide sufficient information to estimate odds ratio (OR) and their 95% confidence intervals (CIs). The exclusion criteria were as the follows: (1) non-comparative studies, reviews or meta-analysis; (2) studies in which necessary data were not provided; (3) for overlapped studies, the studies with low quality were excluded.

### Data extraction

Two investigators (YL Yang and YG Bian) independently reviewed the eligible studies and extracted the data. Disagreements were resolved by discussion among all authors. Data extraction focus on (1) general information including name of first author, publication year, country, research type (randomized controlled trial (RCT) or retrospective study), sample size; (2) patients factor including age, the percentage of emphysema, cavitation present, the lesion size and lesion deep in both case and control group; (3) the technology factor including needle calibers, type of ABP (clotted or non-clotted procedure).

### Quality assessment

The Cochrane Collaborations tool for assessing risk of bias was used to assess the quality of the methodology of RCTs. randomisation, allocation concealment, outcome data, and selective outcome reporting and other sources of bias was assessed. The quality of study could be classified as three categories: low, high or unclear [9]. For non-RCTs, Newcastle–Ottawa scale (NOS) was used [10]. Studies with five or more stars were defined as high-quality studies. Quality assessment was performed by two investigators (Yanlong Yang and Yungui Bian) independently. Disagreements were resolved by discussion.

### Statistical analysis

The odds ratios (ORs) were used to compare dichotomous variables. All outcomes were reported with 95% CIs. Heterogeneity between studies was detected by the Q test and the I^2^ metric (no heterogeneity: I^2^ = 0–25%, moderate heterogeneity: I^2^ = 25-50%; large heterogeneity: I^2^ = 50-75%; and extreme heterogeneity: I^2^ = 75-100%). The fixed-effect model (the Mantel Haenszel method) was only used for studies with no heterogeneity (I^2^ = 0–25%) [11, 12]. Otherwise, random effect model (DerSimonian and Laird) analysis was conducted for I^2^ > 25% [13]. Subgroup analysis according to the emphysema status was done to explore whether the efficacy of ABP could be affected by emphysema. Also, other subgroup analyses including research type (RCT or retrospective study), and type of ABP (clotted or non-clotted) were done to detect potential source of heterogeneity and bias in different subgroups. Publication bias was assessed by the method reported by Begg [14]. Except for the P value for heterogeneity test with a significant level at 0.10, the P values for other analysis < 0.05 was considered statistically. Statistical analysis was performed using Review Manager Version 5.3 (Cochrane Collaboration, Oxford, UK) (The Cochrane Collaboration, Oxford, London, UK) and the STATA software version 11.2 (StataCorp, College Station, Texas, USA).

## Results

### Eligible studies

**Fig. 1:**
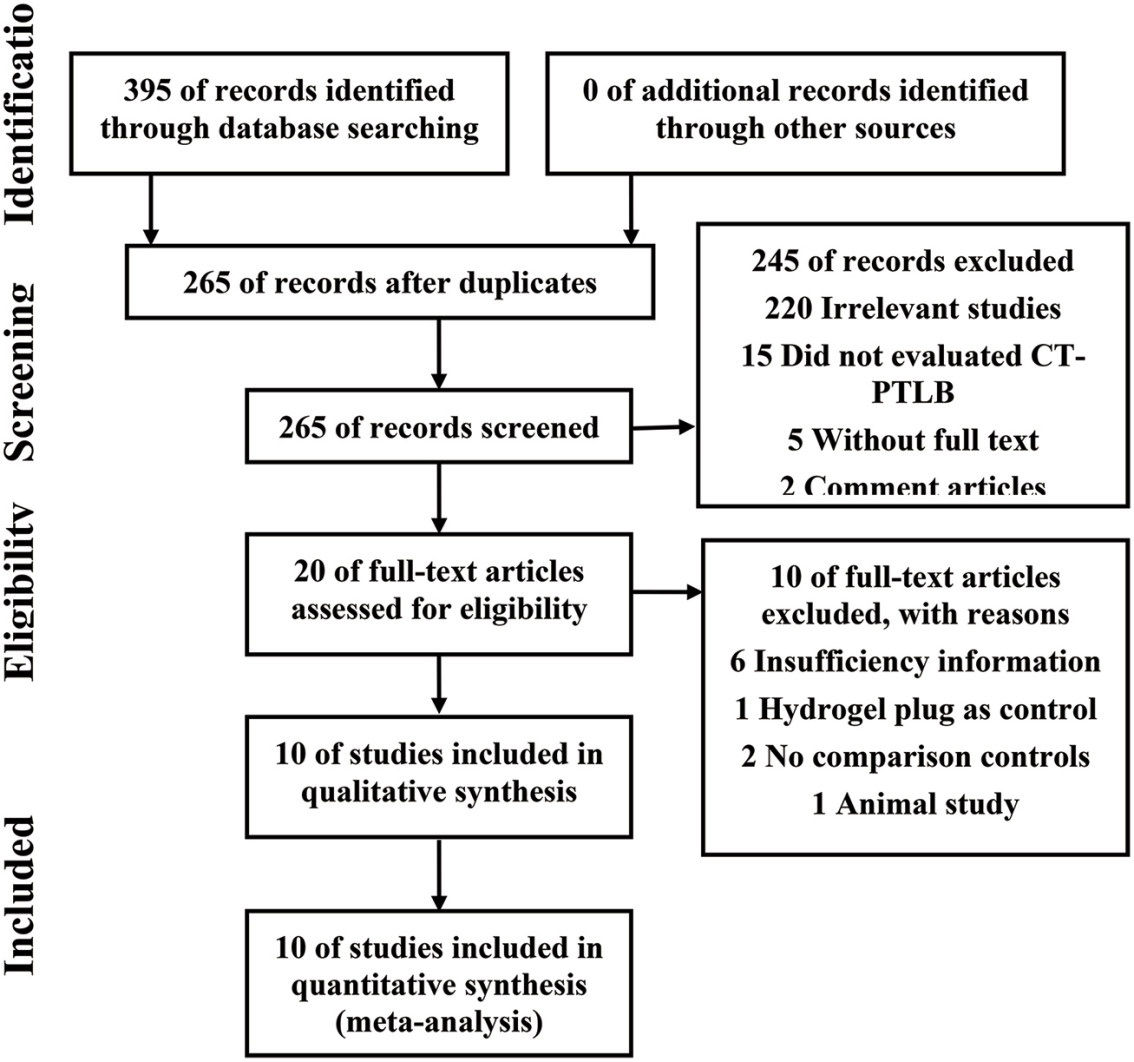
Flow diagram of studies selection procedure

The initial research yielded 265 potential articles, after reading the titles and abstracts,245 studies were excluded because they were obviously irrelevant to our study design. The remaining 20 studies with full texts were carefully assessed for eligibility. As a results, 10 studies were excluded for following reasons: six studies did not provided the sufficient information for us to extract the data, one study choose hydrogel plug as control, two studies were non-comparative studies, one study was conducted in animal. At last 10 studies with 3874 patients (1904 in ABP group and 1970 in control group) [7, 8, 15–22] were included in our studies (Fig. 1).The main characterizes of included studies were summarized in Table 1. In terms of quality, among four RCT studies, One study have low risk of bias, two studies showed unclear risk of bias and two studies showed high risk of bias. The remaining 6 non-RCTs all scored highly (with five stars or more) by NOS (Table S1 and S2).

**Table 1:** Main characteristics of all studies included in the meta-analysis

Author, year, ref	Country	Research type	No.(Case/control)	Age	needle calibers	Procedure	Emphysema (%)	Lesion size	Lesion deep	Cavitation(%)
Bourgouin [15]	USA	RCT	52/88	NR	19G Guide,22G Biopsy	clotted	19.9/ 21.7	3.5 vs. 3.3	0–5:82.9%:>5:17.1%	52/88
Herman [8]	Canada	RCT	46/47	63.2/64.7	19G Guide,22G Biopsy	clotted	NR	3.3/3.9	NR	46/47
Lang [16]	USA	RCT	50/50	51(27–78)	19GGuide, 20/22G Biopsy	Clotted	38	2.2(0.8–5.2)	3.6(1–8)	3
Malone [7]	Canada	RCT	123/119	65 ± 12/66 ± 14	17/19GGuide,18/20G Biopsy	clotted	27.6/31.1	2.2 ± 1.3/2.3 ± 1.5	3.2 ± 1.9/2.9 ± 1.9	123/119
Graffy [18]	USA	Rep	482/352	66.2 ± 10.8/63.4 ± 12.5	19G Guide, 20/21G Biopsy	Clotted	41.50	NR	2.9 ± 1.8/2 ± 1.6	482/352
Clayton [17]	USA	Rep	245/189	67 ± 12.6/66 ± 13.7	19G Guide, 20/22/23G Biopsy	nonclotted	47/49	2.3 ± 1.3/2.3 ± 1.1	NR	245/189
Perl [19]	Germany	Rep	419/449	63.9(16–95)	13,15,17,19G	Non-clotted	NR	NR	NR	419/449
Turgut [20]	Turkey	Rep	91/171	62.6 ± 10.4/59.3 ± 11.7	20G	Non-clotted	8.7 /7.6	3.2(2.0–4.4)/3.0(2.0–3.9)	NR	91/171
Liu [22]	China	Rep	79/55	NR	19G Guide, 20G Biopsy	Non-clotted	40.5/23.6	NR	NR	79/55
Duignan [21]	Ireland	Rep	259/393	68.4 ± 11.6/68.1 ± 12.7	19G Guide, 20G Biopsy	Non-clotted	18.5/7.9	NR	3.0 ± 2.1/2.3 ± 1.9	259/393

*Note* RCT, randomized controlled trial; Rep, retrospective study; NR, not report

ABP reduced the pneumothorax rate when compared with control groups.

**Fig. 2:**
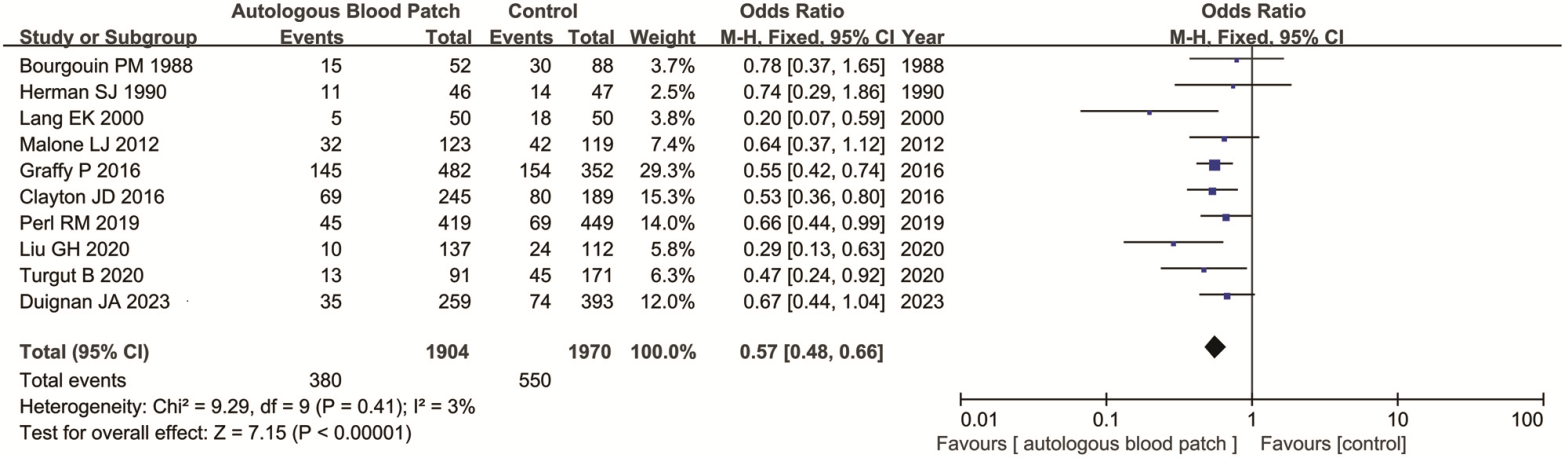
Forest plot of the association between ABP use and pneumothorax rate after CT-PTLB

All included 10 studies including 3874 patients (1904 cases and 1970 controls) evaluated the relationship between ABP and pneumothorax rate. The pooled analysis suggested that ABP was significantly associated with lower pneumothorax rate when compared with control (incidence: 20.0% vs. 27.9%, OR = 0.57, 95% CI = 0.48–0.66, *P* < 0.001) (Fig. 2; Table 2).

**Table 2:** Main meta-analysis results of ABP procedure in patients receiving CT-PTLB

Outcome of interest	No. of studies	No. of patients	OR (95% CI)	p-Value	I^2^(%)	P
**Pneumothorax overall**	10	1904/1970	0.57(0.48,0.66)	< 0.0001	3	0.41
Subgroup 1 Emphysema						
Present	4	287/213	0.37(0.19,0.72)	0.004	47	0.13
Absent	4	505/420	0.51(0.33,0.79)	0.002	28	0.25
Subgroup 2 research type						
RCT	4	271/304	0.58(0.35,0.95)	0.03	37	0.19
Retrospective study	6	1633/1666	0.56(0.47,0.67)	< 0.0001	0	0.49
Subgroup 3 type of ABP						
Clotted	4	271/304	0.58(0.35,0.95)	0.03	37	0.19
Non-clotted	6	1633/1666	0.56(0.47,0.67)	< 0.0001	0	0.49
**Chest drain insertion overall**	9	1485/1521	0.47(0.34,0.65)	< 0.0001	9	0.36
Subgroup 1 research type						
RCT	4	271/304	0.51(0.27,0.95)	0.03	0	0.64
Retrospective study	5	1214/1217	0.50(0.29,0.84)	0.009	43	0.13
Subgroup 2 type of ABP						
Clotted	4	271/304	0.51(0.27,0.95)	0.03	0	0.64
Non-clotted	5	1214/1217	0.50(0.29,0.84)	0.009	43	0.13

**Fig. 3:**
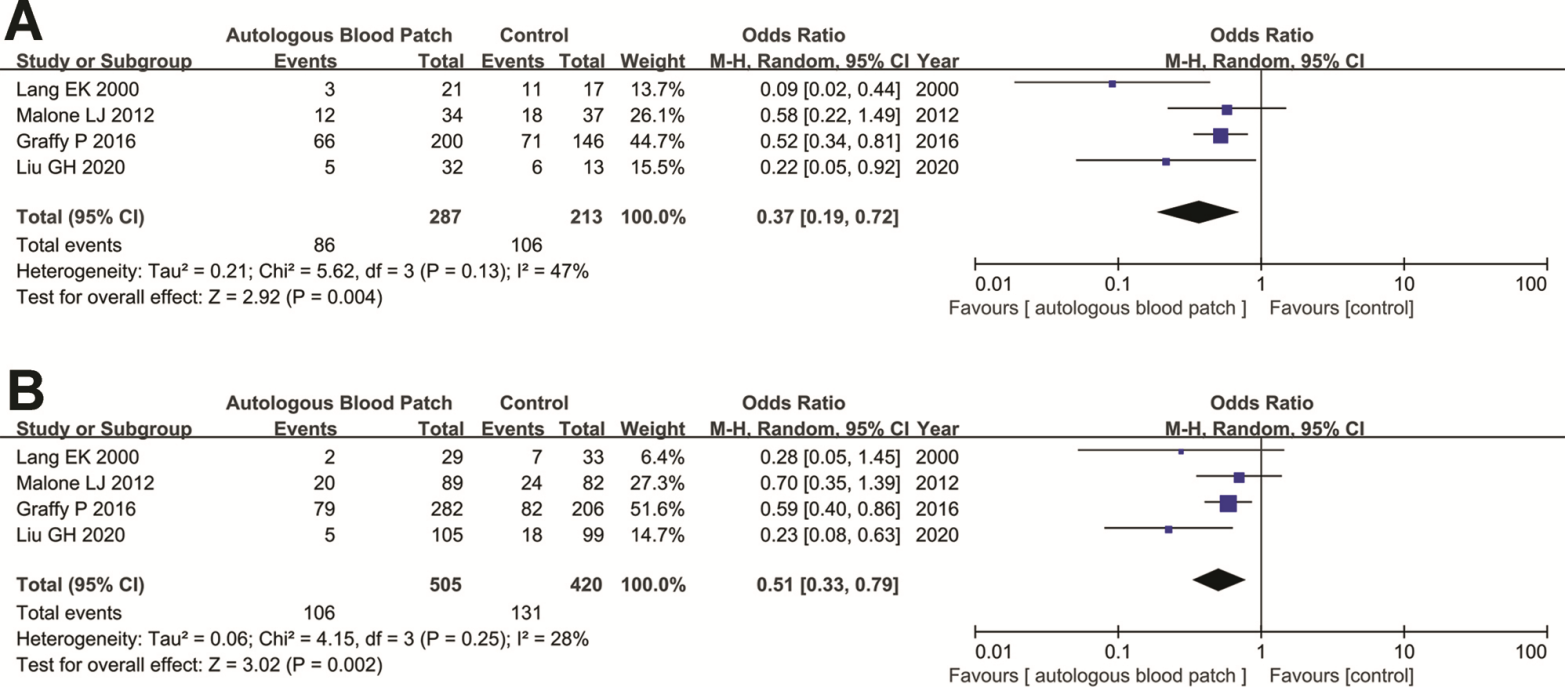
Forest plot of the association between ABP use and pneumothorax rate in patients with emphysema (**A**) and without emphysema (**B**) after CT-PTLB

As patients with emphysema were more easily to develop pneumothorax after CT-PTLB, we collected datasets to evaluate whether ABP could also reduce pneumothorax incidence in patients with emphysema. Finally, four studies evaluated the role of ABP in patients with and without emphysema. The pooled analysis suggested that although the incidence of pneumothorax in patients with emphysema was higher than in those without emphysema, the use of ABP could reduce pneumothorax incidence in not only patients without emphysema (incidence: 20.1% vs. 31.2%, OR = 0.51, 95% CI = 0.33–0.79, *P* = 0.002) (Fig. 3B) but also in patients with emphysema (incidence: 30.0% vs. 49.8%, OR = 0.37, 95% CI = 0.19–0.72, *P* = 0.004) (Fig. 3A).

Subgroup analysis according to study type was done. We found the pooled result of both 4 RCTs and the remaining 6 retrospective studies showed the use of ABP could significantly reduce the incidence of pneumothorax (for RCTs, incidence: 23.2% vs. 34.2%, OR = 0.58, 95% CI = 0.35–0.95, *P* = 0.005; for retrospective studies, incidence: 19.4% vs. 26.8%, OR = 0.56, 95% CI = 0.47–0.67, *P* < 0.001)(Table 2).

ABP technique applied in our study could be classified as clotted and non-clotted blood. The studies applied clotted blood were all RCTs and the remaining studies applied non-clotted blood were all retrospective studies, as a consequence, the result was the same as in the subgroup analysis according to study type, both clotted and non-clotted blood technique could reduce the incidence of pneumothorax after CT-PTLB (Table 2).

ABP reduced the chest drain insertion rate when compared with control groups.

**Fig. 4:**
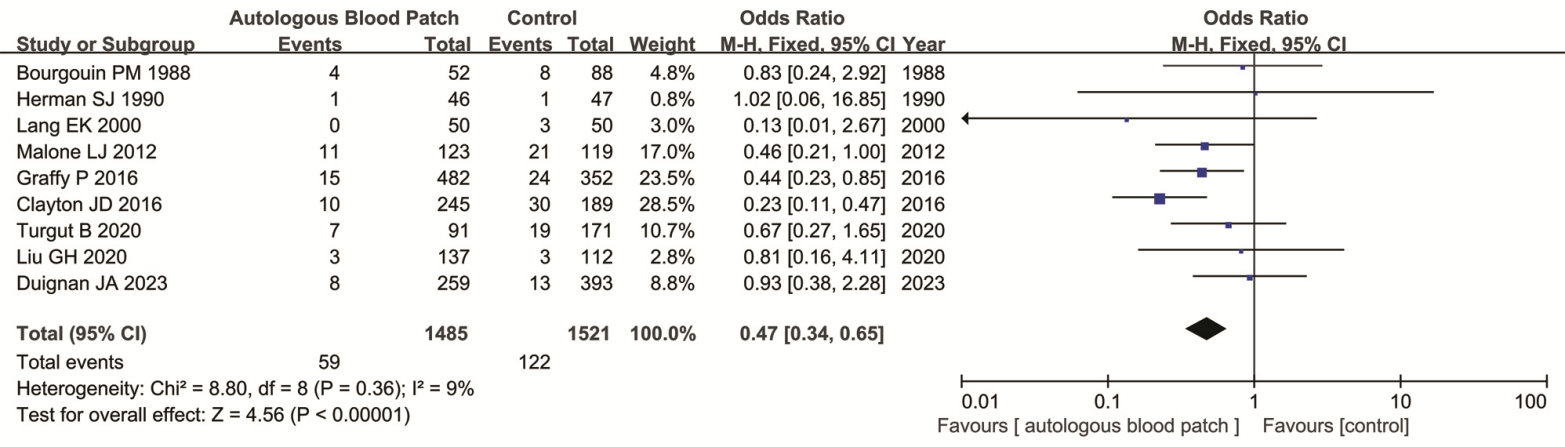
Forest plot of the association between ABP use and chest tube insertion rate after CT-PTLB.

Nine studies with 3006 patients (1485 cases and 1521 controls) evaluate the relationship between ABP and the chest tube insertion rate. The patients with ABP was associated with lower chest tube insertion rate when compared with control groups (4.0% vs. 8.0%, OR = 0.47, 95% CI = 0.34–0.65, *P* < 0.001) (Fig. 4). Subgroup analysis by study type (RCTs and retrospective studies) and type of ABP technique (clotted and non-clotted blood patch) all found significantly reduced chest tube insertion rate in ABP group when compared with control group (Table 2).

### Heterogeneity

No large heterogeneity (I^2^ ≥ 65%) was detected in the all analysis. In pneumothorax rate analysis, we found moderate heterogeneity in the subgroup analysis according to emphysema status (I^2^ for absent = 28%, I^2^ for present = 47%), research type (I^2^ for RCT = 37%) and type of ABP (I^2^ for clotted = 37%). In chest drain insertion rate analysis, studies with RCT and clotted ABP showed moderate heterogeneity (I^2^ = 43).As a result, random effect model (DerSimonian and Laird) analysis was applied. Except this, no significant heterogeneity was detected in other comparisons and the fixed-effect model (the Mantel Haenszel method) was used.

Sensitivity analysis.

**Fig. 5:**
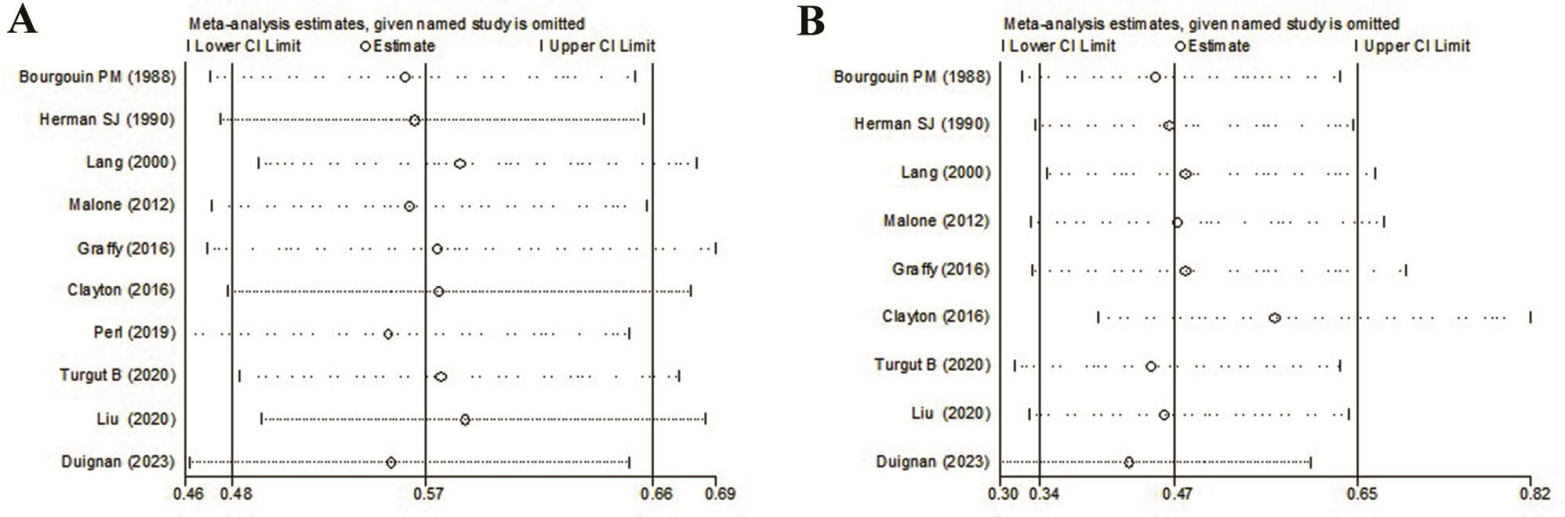
Sensitivity analyses omitting one study each time on the influence of ABP use and pneumothorax rate (**A**) and chest tube insertion rate (**B**) after CT-PTLB

In the sensitivity analysis, the influence of each study on the pooled OR on pneumothorax rate and chest tube insertion rate was examined by repeating the meta-analysis while omitting each study one at a time. The analysis results suggested that no individual study significantly affected the pooled OR, suggesting our result was robust and reliable (Fig. 5A and B).

**Fig. 6:**
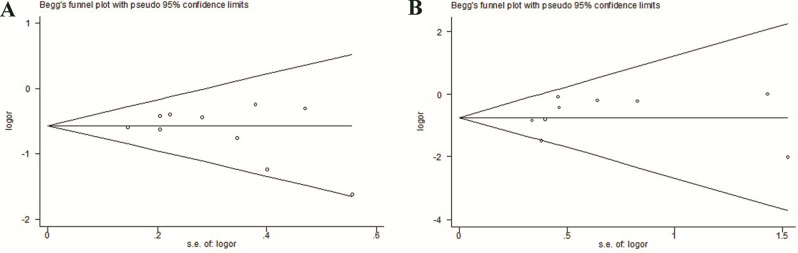
The funnel plot of the meta-analysis of the impact of ABP on pneumothorax (**A**) and chest tube insertion rate (**B**) in patients after CT-PTLB.

### Publication bias

No significant bias was indicated by the Begg’s and Egger’s test (pneumothorax rate: P_Begg_ = 0.47, P_Egger_ = 0.32; chest tube insertion rate: P_Begg_ = 0.39, P_Egger_ = 0.18).

## Discussion

The current study demonstrates the use of ABP was effective technique in reducing the pneumothorax and chest tube insertion rate after CT-PTLB. This is the first study that systematically reviewed the literature for the efficacy of ABP for patients following CT-PTLB. Both overall analysis and all subgroup analysis all showed this significant association.

The use of ABP was first reported by McCartney et al. in 1974. They used dogs as experimental model. The dogs were divided into 3 groups: blood patch, gelfoam and controls. They found after 1 week, a 2–3 mm gray plaque was found in pleural surface of biopsy site in ABP group. Microscopic examination suggested injected blood or fresh hemorrhage filled with needle tract. At 4 weeks, no fresh hemorrhage, inflammatory reaction or fibrosis was observed. In gelfoam group, acute inflammation was observed at 1 week and disappeared at 3 weeks, and followed by local fibrosis at 4 weeks. In control group, hemorrhage and adjacent edema along the needle tract was still exist throughout 4 weeks period. This study demonstrated that ABP paly an equal role in sealing needle tract and pleural. However, ABP showed lower pleural reaction and was easy handling and sterility maintaince [23]. Followed this pre-experimental study, a series of research was done to evaluated the efficacy of ABP in patients receiving CT-PTLB. We collected the existed studies and pooled their result by meta-analysis, and we concluded the use of ABP was effective technique in reducing the pneumothorax and chest tube insertion rate after CT-PTLB.

Four studies also evaluated the ABP in CT-PTLB were not included in our analysis because the study design was not in corroding with our inclusion criteria. The study by Maybody et al. conducted a RCT to compare the efficacy of ABP and commercial hydrogel plug. They concluded ABP is noninferior to a hydrogel plug regarding the rate of pneumothorax after CT-PTLB. However, ABP has several advantages over hydrogel plug: it is essentially free; it does not require a specific introducer needle type, gauge, or length; it can be deployed for lesions closer than 1.5 cm to the pleura; and it is proven to be absorbed shortly after deployment [6]. In the study by Wagner et al., they applied ABP only after pneumothorax complicating CT-PTLB, they also found ABP reduces the need for chest tube placement and hospital admission in patients with pneumothorax complicating CT-PTLB [24]. Another study by Zlevor found parenchymal blood patching during introducer needle withdrawal decreased complications requiring intervention. Salvage pleural blood patching reduced the frequency of chest tube placement for pneumothorax [25]. All these studies we did not included in our analysis confirmed ABP was an efficacy technique in reducing pneumothorax complicating CT-PTLB, which was consistent with our conclusion.

Another issue was the ABP technique itself. The first question was choosing clotted or non-clotted ABP. Our analysis suggested both clotted and non-clotted ABP subgroups showed significant reduced pneumothorax and chest tube placement rate after CT-PTLB. However, clotted ABP technique was more complicated when compared to non-clotted ABP. If non-clotted ABP could achieve the same result, it’s easier to choose non-clotted ABP, However, no such kind of comparison to evaluate this question. Another question was extrapleural or intraparenchymal ABP, which is better. Two studies (one RCT and another retrospective study) by Tu¨rk answered this question that use of extrapleural along with intraparenchymal ABP significantly decreased the pneumothorax rate during biopsy procedure and the intervention rate compared to IAPBI-alone [26, 27]. However, more studies were warranted to confirm this issue.

### Some limitations should be acknowledged

Firstly, RCT was limited. Although 4 RCTs were included, these RCTs were published before 2012, and the sample size was not large enough and potential risk of bias existed in these RCTs. As a result, more rigorously designed RCTs were look forward to draw a more sounded conclusion.

Secondly, although our analysis showed no heterogeneity, potential heterogeneity may exist. Pneumothorax may affected by many factors such as emphysematous lungs, larger calibre guide/needles, bulla crossed, fissure crossed, multiple pleural punctures, smaller lesions, lesions without pleural contact and deeper lesions et al. However, not all studies reported these factors, and we could not conduct subgroup analysis to identify this factor.

At last, potential publication biases may exist. Articles were not written in English and Chinese and studies failed to get published because of negative or null results cannot be identified in our literature search and thus were not included in this analysis. In addition, some reports did not provide sufficient data were also excluded from our analysis.

In conclusion, our study indicated that the use of ABP was effective technique in reducing the pneumothorax and chest tube insertion rate after CT-PTLB. ABP have many advantages such as essentially free and easy to manipulate. With the limitations, heterogeneities, and bias of meta-analysis, our conclusions in this study need to be interpreted with caution. Future large RCTs with rigorously designed methodology are warranted to confirm our results.

### Electronic supplementary material

Below is the link to the electronic supplementary material.

Supplementary Material 1

## Data Availability

All data generated or analyzed during this study are included in this article.
