# Supplementary material for: Efficacy of autologous blood patch injection for pneumothorax rate after CT-guided percutaneous transthoracic lung biopsy: a systematic review and meta-analysis
Source: J Cardiothorac Surg. 2024 Jun 14;19:332. doi: 10.1186/s13019-024-02781-0 (PMC11177476; doi:10.1186/s13019-024-02781-0)
Supplement: Supplementary file 1 — Supplementary Material 1 [file 13019_2024_2781_MOESM1_ESM.docx]

Table S1 Quality assessment of RCTs with Cochrane’s Collaboration Tool

| author, year | Random sequence generation（selection bias） | Allocation concealment（selection bias） | Blinding of participants and personnel (performance bias) | Blinding of outcome assessment (detection bias) | Incomplete outcome data (attrition bias) | Selective reporting (reporting bias) | Other bias |
| --- | --- | --- | --- | --- | --- | --- | --- |
| Bourgouin PM,1988 | High risk | High risk | Low risk | Low risk | Low risk | Low risk | Low risk |
| Herman SJ,1990 | High risk | Low risk | Low risk | Low risk | Low risk | Low risk | Unclear risk |
| Lang,2000 | Unclear risk | Unclear risk | Low risk | Low risk | Low risk | Low risk | Low risk |
| Malone,2012 | Low risk | Low risk | Low risk | Low risk | Low risk | Low risk | Low risk |

Table S2.Quality assessment of non-RCTs with Newcastle-Ottawa Scale

| Author | Year | Selection | Comparability | Outcome |
| --- | --- | --- | --- | --- |
| Graffy | 2016 | ★★★ | ★★ | ★★ |
| Clayton | 2016 | ★★★ | ★ | ★★ |
| Perl | 2019 | ★★★ | ★ | ★★ |
| Turgut B | 2020 | ★★★ | ★★ | ★★ |
| Liu | 2020 | ★★★ | ★★ | ★★★ |
| Duignan | 2023 | ★★★ | ★★ | ★★ |
